# Changing genetic paradigms: creating next-generation genetic databases as tools to understand the emerging complexities of genotype/phenotype relationships

**DOI:** 10.1186/1479-7364-8-9

**Published:** 2014-05-22

**Authors:** Bruce Gottlieb, Lenore K Beitel, Mark Trifiro

**Affiliations:** 1 Lady Davis Institute for Medical Research, 3755 Côte Ste Catherine Road, Montreal, QC H3T 1E2, Canada; 2 Segal Cancer Centre, Jewish General Hospital, 3755 Côte Ste Catherine Road, Montreal, QC H3T 1E2, Canada; 3 Department of Human Genetics, McGill University, Montreal, QC, Canada; 4 Department of Medicine, McGill University, Montreal, QC, Canada

**Keywords:** Human genetic variation, Next-generation sequencing, Genotype to phenotype relationships, Next-generation genetic databases

## Abstract

Understanding genotype/phenotype relationships has become more complicated as increasing amounts of inter- and intra-tissue genetic heterogeneity have been revealed through next-generation sequencing and evidence showing that factors such as epigenetic modifications, non-coding RNAs and RNA editing can play an important role in determining phenotype. Such findings have challenged a number of classic genetic assumptions including (i) analysis of genomic sequence obtained from blood is an accurate reflection of the genotype responsible for phenotype expression in an individual; (ii) that significant genetic alterations will be found only in diseased individuals, in germline tissues in inherited diseases, or in specific diseased tissues in somatic diseases such as cancer; and (iii) that mutation rates in putative disease-associated genes solely determine disease phenotypes. With the breakdown of our traditional understanding of genotype to phenotype relationships, it is becoming increasingly apparent that new analytical tools will be required to determine the relationship between genotype and phenotypic expression. To this end, we are proposing that next-generation genetic database (NGDB) platforms be created that include new bioinformatics tools based on algorithms that can evaluate genetic heterogeneity, as well as powerful systems biology analysis tools to actively process and evaluate the vast amounts of both genomic and genomic-modifying information required to reveal the true relationships between genotype and phenotype.

## Introduction

The problem of understanding the relationships between genotype and phenotype has become very much more complicated with the explosion of genetic information produced by next-generation sequencing (NGS). This information has greatly complicated not only our ability to understand complex traits, but also our understanding of monogenic traits is no longer quite so straight forward. Indeed, recent articles have suggested the need to develop new approaches to come to grips with the ever-expanding complexity of genotype/phenotype relationships, such as ‘systems genetics’ [1] and ‘particle genetics’ [2].

**Figure 1:**
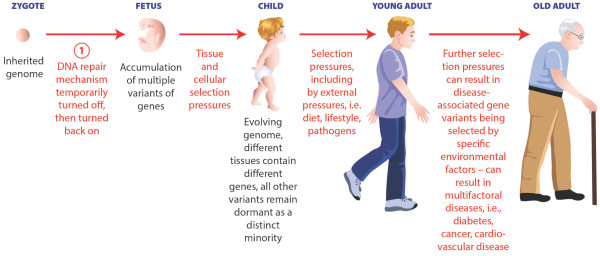
**Factors that can affect an individual's genome from conception to death.** (1) We have postulated that somatic mutations may occur during embryogenesis, and are then selected for later in life, to emphasize both the importance of identifying mutations early in development and the role of selection in determining phenotype.

However, perhaps the most confusing from a ‘traditional’ genetics standpoint has been the revelation of unexpected amounts of genetic variation in normal individuals, e.g., through the 1000 Genomes Project Consortium [3,4] (http://www.1000genomes.org), and The Cancer Genome Atlas (http://www.cancergenome.nih.gov) projects. Further, multiple sequence comparisons both between and within an individual's tissues have revealed extensive inter- and intra**-**tissue genetic heterogeneity [5-7]. These discoveries have raised some fundamental questions about our most basic genetics assumptions, among which are the following: (i) Can genetic studies still rely on a *unique* DNA or RNA sequence derived from blood or diseased tissue to determine phenotype?; (ii) Does a definitive and practical human genome reference sequence really exist, or at least can the reference sequence adopted by the NCBI (RefSeqGen) be practically useful in determining genotype/phenotype relationships?; and (iii) Does genetic heterogeneity in normal and diseased tissues imply that in certain tissues an individual's genome will naturally undergo somatic changes from conception to death as suggested in Figure 1. In particular, newly revealed genetic heterogeneity data could help explain the long observed, but poorly understood concepts of variable expressivity and reduced penetrance. Traditionally, their effects on phenotypic differences have been considered to be relatively insignificant, particularly so for variable expressivity. To further complicate matters, phenotypic variations have been found, where identical gene alterations have been associated with (i) considerably different disease phenotypes, e.g., in phenylalanine hydroxylase deficiency (PAH) [8], or (ii) in a more extreme manner in the androgen receptor (AR) gene, with both androgen insensitivity syndrome (AIS) and prostate cancer [9].

**Figure 2:**
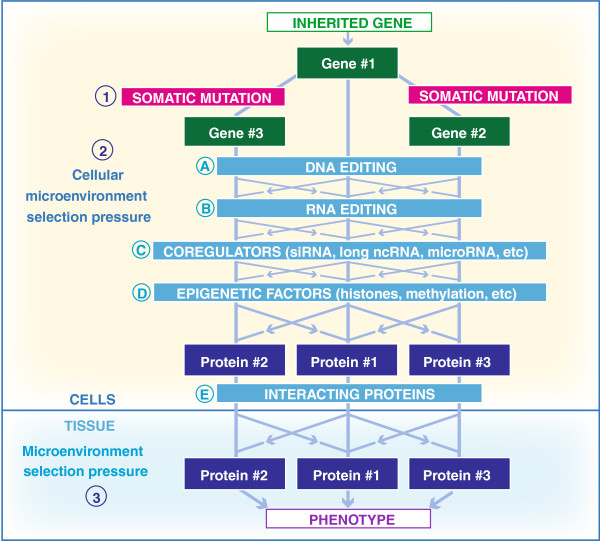
**Phenotypic modifying factors.** (1) Somatic mutations can include both single nucleotide variants and structural alterations such as copy number variations that can then result in somatic and clonal mosaicism. (2) Cellular microenvironment selection pressure can work at the (i) DNA level, i.e., due to somatic mutations or (A) DNA editing; (ii) RNA level, i.e., due to (B) RNA editing, (C) interacting RNAs, or (D) epigenetic factors, etc.; or (iii) protein level, i.e., due to (E) protein-protein interactions. (3) Tissue microenvironment selection pressure can select a different protein product. *Crossing arrows* reflect the fact that selection can go in either direction.

In addition, there has also been an increase in the discovery of significant phenotype-modifying events, including epigenetic modifications, RNA editing, and protein interactions that can clearly influence transcriptional and non-transcriptional events involved in determining the phenotype. Thus, these complex influences are also likely to render our traditional understanding of the relationship between genotype and phenotype problematical. Further, a recent review of genotype/phenotype dissociation that discussed the possible molecular basis of reduced penetrance in human inherited disease, highlighted 12 molecular events that can influence reduced penetrance [10], some of which are also likely involved in situations of variable expressivity. In Figure 2, we have suggested a model that incorporates some of these processes, and how they might influence phenotype, with special emphasis on the influence of intra-organismal and intra-tissue genetic heterogeneity. Traditionally, genetic databases have been the tools of choice in determining genotype/phenotype relationships; however, in their present form, they are totally inadequate to deal with these issues. Therefore, we are suggesting that it is time to create next-generation genetic databases (NGDB) that will be able to incorporate and analyze all of the factors that can contribute to the dissociation of genotype from phenotype, including those that may contribute to reduced penetrance and variable expressivity.

### Factors that have been shown to influence phenotype

#### Somatic mutations that result in intra-organismal and intra-tissue genetic heterogeneity

Until recently, it has been assumed that somatic mutations are almost exclusively associated with cancers and are uniform within an individual neoplasm. However, different sets of somatic mutations have been found within a single individual's cancer tissues, as in a recent study of primary high-grade serous ovarian cancers that revealed a considerable amount of intra-tumor genetic heterogeneity [11].

Somatic sequence variants in normal tissues have also been examined in relation to oncogenesis. One study concluded that somatic sequence variants in normal cell populations could be the earliest stage of oncogenesis [12]. Evidence that altered mammary gland development and predisposition to breast cancer is due to *in utero* exposure to endocrine disruptors has suggested that selection of cells with different phenotypic properties, presumably as a result of very early somatic mutations, may take place at the very earliest stages of breast tissue development [13]. Thus, we may need to reconsider whether accumulation of a critical number of oncogenic mutations, e.g., the buildup of driver somatic mutations, is the reason that many cancers occur later in life. Rather, it has been proposed that while the genetic origins of cancer may occur early in fetal development, *later selection pressure* could explain the relationship between aging and cancer [14]. Interestingly, a possible mechanism to produce very early somatic mutations, namely the temporarily deferring of the repair of DNA lesions encountered during tissue replication, that has been termed damage bypass, has been identified as responsible for somatic hypermutation of the immunoglobin gene [15]. Regardless of which oncogenesis hypothesis is eventually proven, the implications for construction of NGDB for cancers is likely to be profound, as NGDBs will need to consider incorporating sequence data from much earlier stages in a tissue development, particularly from tissues that have the potential to become cancerous. Obviously, the ability to do so at the moment is not practical, but it is possible to envision that in the future, new micro-sampling techniques, together with the continued dramatic decline in the cost of NGS, will make such an approach much more realistic.

In addition, as specific tissues are being sequenced routinely, the number of other diseased tissues in which somatic mutations have been found has increased considerably [16]. More detailed studies have also reported somatic mosaicism in a number of other conditions, including the Proteus syndrome [17] and hemimegalencephaly [18].

Further, a study of copy-number variants (CNVs) in somatic human tissues revealed a significant number of intra-individual genomic changes between tissues [19]. Other studies of chromosomal abnormalities, including CNVs have revealed clonal mosaicism associated with aging and cancer [14], as well as related it to a higher risk of hematological cancer [20].

#### DNA editing

At the present state of our knowledge, this process is still considered to be extremely rare and of little phenotypic significance [21].

#### RNA editing

Recent, though controversial, evidence has suggested that RNA editing occurs more frequently than previously thought [22,23], although questions of how common it actually is in normal tissues and the validity of the original report have arisen [24-26]. However, there do appear to be cases where modifications of disease phenotypes are related to RNA editing [27,28].

#### Coregulators: non-coding RNAs

In recent years, non-coding RNAs (ncRNA) have been found to play an important role in the phenotypic expression of the transcribed genomic output. This family of untranslated RNAs includes small nucleolar RNAs (snoRNAs), which facilitate mRNA splicing, regulate transcription factors, and repress gene expression [via microRNAs (miRNAs)]. Small nuclear RNAs (snRNAs) that alter cellular proliferation and apoptosis by means of small interfering RNAs (siRNAs) have also been identified [29]. Long non-coding RNAs (lncRNAs) have also been identified as possible regulators of gene transcription and expression. Thus, the use of NGS to infer transcript expression levels in general, specifically *via* ncRNAs, is becoming increasingly common in molecular and clinical laboratories [30]. Therefore, it is not surprising that ncRNAs have been implicated as being responsible for a number of disease phenotypes [31].

#### Epigenetic factors

Epigenetics describes chromatin-based events that regulate DNA-templated processes and result in stable reprogramming of gene expression in response to transient external stimuli. Primary epigenetic factors include modifications to DNA and histones that are dynamically added and removed by chromatin-modifying enzymes in a highly regulated manner. Epigenetic mechanisms identified include DNA methylation, phosphorylation, ubiquitylation, sumoylation, RNA interference, and histone variance. Further, such epigenetic modifications play a critical role in the regulation of DNA-based processes such as transcription, DNA repair and replication, which can affect phenotype expression. Thus, abnormal expression patterns or genomic changes in chromatin regulators can have profound effects on human disease processes [32]. Indeed, epigenetics is considered a unifying factor in the etiology of some complex traits [33].

#### Regulators and other types of interacting proteins

Over the past few years, phenotypic expression has also found to be influenced by interacting proteins. Alterations in the interacting surfaces of a specific molecule [34] or the interacting proteins themselves can result in faulty protein-protein interactions and contribute to a disease phenotype [35].

#### Selection pressure by cellular and tissue microenvironments

It has been proposed that tumor morphology and phenotype are driven by selective pressure from the tissue microenvironment [36,37]. This hypothesis has been expanded to include other genetically determined diseased and non-diseased phenotypes [38]. The ability to perform ultra-deep sequencing using next-generation sequencers has revealed many more variants of a gene within tissues and thus the possibility that evolution at the tissue level contributes to disease phenotypes such as cancer [37,38].

### Genotype/phenotype disconnects and possible mechanisms

In light of all the potential phenotype-modifying factors (Figure 2), which are generally not documented in traditional genetic databases, it is easy to understand why such databases, in their attempt to link a defined genotype with a specific phenotype, tend to avoid commenting on genotype/phenotype disconnects, due to the lack of information regarding the mechanisms that could produce such effects. However, a recent review highlighted the importance of understanding these disconnects, with over 650 references cited in proposing 12 molecular mechanisms to explain reduced penetrance [10]. Similarly, a number of possible mechanisms have been suggested to explain variable expressivity, e.g., somatic mosaicism [39], modifier genes [40], microRNA [41], epigenetic processes [42], and allelic heterogeneity [43]. Originally, the concept of reduced penetrance was based on studies of well-known genetic conditions in which a family tree predicted a disease phenotype, but this phenotype was not observed. While in most cases, the likelihood of reduced penetrance was small, it did serve a useful purpose in calculating the possibility of an individual having a diseased phenotype. The concept was further expanded when large-scale studies started to record the presence of mutations in specific genes associated with multifactorial diseases, such as cancer, a prime example being the breast cancer BRCA genes. In these cases, predicting penetrance was considered important in assessing the risk of disease. What has further complicated the issue, as we have noted, has been recent data from the 1000 Genomes Project and other large scale sequencing projects, which have reported that normal individuals can contain tens of potentially severe disease-associated alleles [10]. Thus, rather than talk about reduced penetrance of a pathogenic variant in a cohort that is known to express the disease phenotype, we now have to consider why these pathogenic variants are non-penetrant in a significant number of normal healthy individuals.

### Redefining the human genome reference sequence

Clearly, the arrival of relatively inexpensive whole genome sequencing, and the subsequent sequencing of large numbers of non-diseased individuals, has revealed the increasing presence of known disease-associated gene variants within non-diseased individuals. This was initially shown when the first Korean genome sequence was compared to other Asian genomes [44]. More detailed studies found sequence variants in genes associated with specific genetic disorders, in individuals with normal phenotypes. Such examples were recently discovered in a genomic analysis of 10 healthy individuals, where each individual had what was said to be ‘healthy variance’ in 19 to 31 OMIM genes, as they did not exhibit any of the signs, symptoms, or phenotypes of the associated genetic disorders [45]. However, it should be noted that not all sequence variants in OMIM genes are always pathogenic, as has recently been comprehensively reported [10]. Nevertheless, a systematic survey of loss-of-function (LoF) variants identified 26 known and 21 predicted severe disease-causing variants in analysis of 2,951 putative LoF variants obtained from 185 human genomes [46]. What is even more problematic is that our own work has identified specific pathogenic sequence variants in the AR gene in individuals with completely normal phenotypes, i.e., exactly the same AR variants as found in diseased individuals [9].

We believe this data calls into question the validity of our present methods of defining the so-called normal human genome. In particular, normal tissue genotype/phenotype disconnects have clearly created questions regarding the practicality of relying on a single unique reference sequence as the definitive predictor of phenotype. The Human Genome Variation Society (HGVS) nomenclature committee has studied this issue (http://www.hgvs.org/mutnomen/refseq.html) and recommended that the NCBI RefSeqGen be used and that the reference sequence guidelines should follow the Locus Reference Genomic (LRG) sequence format [47], which suggests using a single-file record containing a unique stable reference sequence. These recommendations were appropriate at the start of NGS, when the extent of variance in normal individuals, was relatively unknown. Naturally, we understand that a definitive reference sequence is important in defining exonic, intronic, and other structural parameters of genes. However, the issue of correlating phenotype with a specific sequence has clearly become much more complex.

To deal with this issue, the increasing amount of sequence variability in normal individuals has been incorporated into the latest version of the NCBI RefSeqGen (GRC37p13) (http://www.ncbi.nlm.nih.gov/projects/genome/assembly/grc/human), with the idea that these variants could be used as a contextual filter to determine the relationship between genotype and phenotype. Furthermore, additional tools have been set up to deal with the issue of normal variance, such as considering population-specific references where the major alleles are included at every location, or generating a reference sequence where all the alleles have been identified as part of the common ancestral lineage of modern humans. However, we would argue that just integrating normal human variance, however nuanced, into an overall version of the RefSeqGen fails to deal with the increasing problem of the association of the same gene variant with both normal and diseased phenotypes. Thus, relying solely on a DNA-based reference sequence, however sophisticated, will make it very difficult to distinguish between benign and disease-causing gene alterations, at least in traditional genetic databases, where the phenotypic classification of specific gene variants is based on having a unique reference sequence that is exclusively associated with a normal phenotype.

### Possible organization of next-generation genetic databases

As an overlying principle, NGDBs need to be organized to take into consideration, particularly for multifactorial diseases, the overall genetic *context* of any identified mutation. However, context involves both intra-organismal genetic heterogeneity as well as other phenotype-modifying factors (Figure 2). These modifying factors also need to be considered in the context of ‘pathway analysis’ [48]. In light of the many contextual factors that can affect the genotype/phenotype expression, it seems reasonable that future of locus-specific databases (LSDBs) should be organized to take into account as much specific phenotype information as possible, including genotype-modifying factors, as opposed to most present LSDBs that are primarily genotype centered.

The issue of how to deal with the increasing identification of somatic mutations and intra-organismal genetic heterogeneity also needs to be investigated. Traditionally, somatic mutations have not been associated with databases unless a cancer phenotype was involved. At present, most disease-based databases associated with common multifactorial diseases such as cancer, diabetes and cardiovascular diseases often lack tissue and individual specific data. Indeed, only the COSMIC database [49] lists a comprehensive spectrum of somatic mutations associated with specific tissues and individual samples. Furthermore, currently, there is no description of the germline susceptibility variants found in matching control tissues, therefore making it difficult to draw definitive conclusions as to the significance of many somatic mutations. The situation will become even more complex when inter- and intra-tumor genetic heterogeneity data is added. Clearly, traditional flat-file databases will be unable to deal with such data and what are needed are radically different database structures that include much more powerful analysis tools. In particular, it will be necessary to incorporate complex ‘system analysis tools’ that can analyze the intricate relationships between genotypic and phenotypic ontology [50]. Such analysis tools will need to incorporate extremely powerful knowledge analysis engines, possibly similar in design and organization to those developed by Google and other search engine companies.

These knowledge engines, for ‘systems genetics analysis’, will require the creation of powerful new bioinformatics tools and tremendously expanded database resources, particularly for disease-based databases. In particular, they will be required to analyze integrated *genetic* and *non-genetic* variation across many datasets, from different ethnic sub-groups or geographic populations, with the ultimate goal of integrating all genetic and non-genetic databases for a particular condition, especially if an initial population-based analysis fails to generate any significant insights into genotype/phenotype relationships. At the moment, such a task is clearly far beyond our capabilities; however, initial studies using mice have started to generate the bioinformatics tools and database resources required to create such NGDBs [51]. As NGDBs will include inter- and intra-tissue genetic heterogeneity, one factor that needs to be considered is the importance of quantifying variants that result in genetic heterogeneity, particularly if they are present within individual genes, rather than simply recording their presence. Indeed, we recently analyzed intra-tissue genetic heterogeneity in the AR gene in both cancer and non-cancer tissues taken from breast tumors and quantified AR variants in individual tissue samples using a new NGS technique [52]. Another approach has been to consider what has been termed ‘particle genetics’, where every cell is considered to be genetically unique, using probabilistic trait loci (PTL) to link genomic regions to probabilities of cellular characteristics [2].

**Figure 3:**
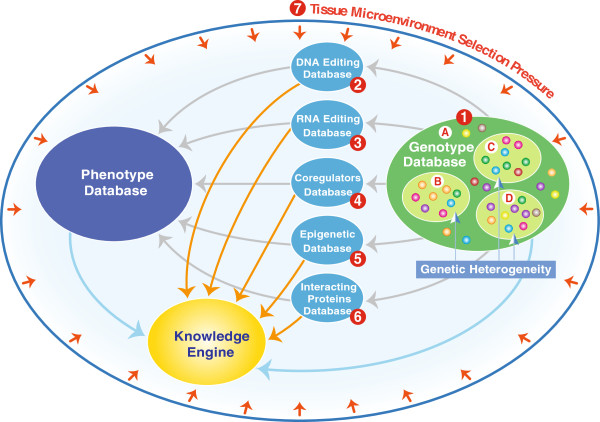
**A model for next-generation genetic databases.** (1) Genotype Database: (A) genetic heterogeneity within blood tissues and (B, C, and D) within other tissues in an organism. Each of the following databases contains specific information associated with phenotype differences: (2) DNA editing database, (3) RNA editing database, (4) Coregulators database, (5) Epigenetic database, and (6) Interacting proteins database. (7) Microenvironment selective pressure for different phenotypes.

Taking all of these factors into consideration, we would propose a NGDB model that integrates separate databases for each of the potential genome-modifying factors, together with a genotype database that incorporates genetic heterogeneity, with all of the individual databases linked to an associated phenotype database, and the data is then processed and analyzed through a very sophisticated knowledge engine (Figure 3).

### Summary of possible actions required to create NGDBs

The following are some of the most significant actions that need to be undertaken in creating NGDBs:

1. Work in conjunction with the 1000 Genomes Project consortium and the Human Variome Project (HVP) to define the limits and significance of normal genome variation.

2. Incorporate individual inter- and intra-individual genetic heterogeneity into NGDBs.

3. Establish guidelines as to the significance of the number of reads needed to confirm a particular variant. Note, that initial NGS sequencing depth started at 4× to 10× coverage and rapidly rose to where 30× to 50× coverage is considered normal. However, recent studies show that increased coverage is likely to result in increased detection of variants [53,54], which in the case of tumor diagnostics coverage has now reached up to 20,000 reads.

4. Determine how the different frequency of occurrence of multiple gene variants within individuals should be incorporated into NGDBs. It should be noted that, at the moment, such frequencies are generally not incorporated into databases, particularly not into LSDBs. It would also clearly help to integrate structural variant data such as CNVs into LSDBs.

5. Incorporate expression data effectively into phenotype data parameters in NGDBs. Note that examples of tissue-specific variations in gene expression have now been reported [55]. In addition, data from the Genotype-Tissue Expression project [56] could be invaluable in determining relationships between tissue gene expression and disease phenotype.

6. Finally, research the bioinformatics and data parameters required to construct NGDBs that can incorporate and analyze all of the above data. To be truly effective, we believe that this effort should involve experts in genetics, bioinformatics, and systems biology-based search and knowledge engines, as well as a worldwide effort to collect genetic variation as for instance, proposed by the HVP.

### Suggestions for future actions to be taken by the HVP

We believe that HVP is an organization that could play a leading role in developing NGDBs first by creating a special committee to look into future genetic database designs to deal with some of the issues raised in this article. Such a committee might include not only nomenclature experts, but also experts in creating both the algorithms required to design the databases, as well as the search and analytical engines. Based on the recommendations of this committee, the HVP could then set up an Institute for Genetic Database Research, which in addition to being responsible for NGDB design, could create a working model of the infrastructure required to run such databases on a worldwide scale. In particular, it will be important to establish a universal design structure so that all NGDBs will have a high degree of compatibility, and we believe that if such a design is coordinated through HVP, which already plays such a role in genetic nomenclature, it is much more likely to be accepted. Finally, in the age of data clouds and sophisticated communication platforms, such an institution need not have a physical structure, but rather could be a virtual institute, that would then allow experts from all over the world to participate.

## Conclusion

For many years, genetics and related medical research have been based on the concept that genetic diseases are the result of alterations to a basically stable human genome that has limited natural variation within individuals, so that single or, in the case of multifactorial diseases, a number of very rare alterations to the human genome are directly responsible for specific diseases. Our initial response to the discovery of increased genetic complexity, particularly in multifactorial diseases, has been to use statistical-based approaches, such as GWAS to try to identify significant rare variants. However, most of these studies have yet to produce the breakthroughs initially predicted, perhaps because they are still analyzing ‘silos of genetic information’ and ignoring the fact that the genomic makeup and phenotypic modifications of every individual are both complex and dynamic. Indeed, the increasing use of NGS, together with more accurate expression and pathway analysis tools, is further broadening our understanding of genotype/phenotype relationships, by revealing that the new genetic landscape is infinitely more complex, not only between individuals, but also within individuals. In such a genetic scenario, multifaceted worldwide NGDBs are likely to be essential tools in our fight to treat genetic-based disease.

## Competing interests

The authors declare that they have no competing interests.

## Authors' contributions

BG conceived and drafted the article, LKB and MT contributed to the discussion of the concepts and ideas presented and helped edit the text. All authors read and approved the final manuscript.
